# The Influence of Environmental Factors on Sleep Quality in Hospitalized Medical Patients

**DOI:** 10.3389/fneur.2014.00267

**Published:** 2014-12-11

**Authors:** Milena Bano, Federica Chiaromanni, Michela Corrias, Matteo Turco, Michele De Rui, Piero Amodio, Carlo Merkel, Angelo Gatta, Gabriella Mazzotta, Rodolfo Costa, Sara Montagnese

**Affiliations:** ^1^Department of Medicine, University of Padova, Padova, Italy; ^2^Department of Biology, University of Padova, Padova, Italy

**Keywords:** hospital, light, noise, sleep, circadian rhythms, internal medicine

## Abstract

**Introduction:** Sleep–wake disturbances are common in hospitalized patients but few studies have assessed them systematically. The aim of the present study was to assess sleep quality in a group of medical inpatients, in relation to environmental factors, and the switch to daylight-saving time.

**Methods:** Between March and April 2013, 118 consecutive inpatients were screened and 99 (76 ± 11 years; hospitalization: 8 ± 7 days) enrolled. They slept in double or quadruple rooms, facing South/South-East, and were qualified as sleeping near/far from the window. They underwent daily sleep assessment by standard questionnaires/diaries. Illuminance was measured by a luxmeter at each patient’s eye-level, four times per day. Noise was measured at the same times by a phonometer. Information was recorded on room lighting, position of the rolling shutters and number/type of extra people in the room.

**Results:** Compliance with sleep-wake assessment was poor, with a range of completion of 2–59%, depending on the questionnaires. Reported sleep quality was sufficient and sleep timing dictated by hospital routine; 33% of the patients reported one/more sleepless nights. Illuminance was generally low, and rolling shutters half-way down for most of the 24 h. Patients who slept near the window were exposed to more light in the morning (i.e., 222 ± 72 vs. 174 ± 85 lux, *p* < 0.05 before the switch; 198 ± 72 vs. 141 ± 137 lux, *p* < 0.01 after the switch) and tended to sleep better (7.3 ± 1.8 vs. 5.8 ± 2.4 on a 1–10 scale, before the switch, *p* < 0.05; 7.7 ± 2.3 vs. 6.6 ± 1.8, n.s. after the switch). Noise levels were higher than recommended for care units but substantially comparable across times/room types. No significant differences were observed in sleep parameters before/after the switch.

**Conclusion:** Medical wards appear to be noisy environments, in which limited attention is paid to light/dark hygiene. An association was observed between sleep quality and bed position/light exposure, which is worthy of further study.

## Introduction

Sleep–wake disturbances are common in medical wards. Disrupted sleep has been correlated with potentially harmful health effects such as increased incidence of cardiovascular disease (1), impaired immune function (2), elevated stress response, attention and memory deficits, depressed mood (3) and, in hospitalized patients, a longer length of hospitalization (4). Hospitalization is a difficult moment for a patient: in addition to the physical illness leading to admission and the psychological stress connected to it, the ward environment can be damaging to the induction and maintenance of a normal sleep-wake cycle. Environmental stimuli are a source of sleep deprivation, with critically ill patients receiving up to 60 interruptions per night (5). Therapeutic and diagnostic procedures, noise and light at night are the main causes for patient’s arousals, contributing to interruptions in their sleep (6, 7). Moreover, patients are taken away from their familiar setting and placed in a new environment, with different and sometimes strong time cues. Spending too much time in bed, being inactive and taking medications can heavily impinge on the sleep-wake cycle, weaken circadian rhythms and lead to learnt insomnia (8).

Light is the main environmental signal (*Zeitgeber*) for synchronizing the circadian clock. Light exposure in the morning/evening has been proven to advance/delay the circadian phase (9). To date, there are no recommended illuminance values to be exposed to, nor has the minimum luminance value been identified that is able to keep the circadian master clock synchronized (10–13). Previous studies have suggested that hospitalized patients might be exposed to too low luminance levels, with little differences in day-night light exposure, which may be insufficient for circadian entrainment and contribute to advanced or delayed rhythms, depending on timing and intensity (14, 15). Noise is another environmental factor that has been shown to disrupt sleep in inpatients, especially in intensive care units (16, 17).

Finally, in healthy volunteers the spring switch to daylight-saving time has been associated with disrupted sleep (18, 19), reduced performance and reduced vigilance (20). The effects of the switch to daylight-saving time in hospitalized medical patients remain largely unknown.

The aim of the present study was to assess sleep quality and sleep timing in a large group of well-characterized medical inpatients, in relation to environmental factors, and the switch to daylight-saving time.

## Materials and Methods

### Study setting and patients

The study was conducted in the internal medicine ward Clinica Medica V of Padua University Hospital. Two double and four quadruple rooms facing South/South-East were utilized, each equipped with a central lighting system, and a headboard lamp controlled by the patient on each bed.

One hundred and 18 consecutive inpatients were screened between 1 March and 21 April 2013, 17 (14%) were excluded because their inpatient stay spread across the switch to light saving time (30 March 2013), 2 (1.7%) because their inpatient stay was less than 48 h. Ninety-nine inpatients (62 before and 37 after the switch to daylight-saving time; mean age 76 ± 11 years, 55 males; average length of hospitalization: 8.4 ± 6.7 days) were enrolled; reasons for admission are detailed in Table 1. Patients were qualified as sleeping near (distance <1 m) or far (distance >3 m) from the window. Data were collected daily on the administration of sleeping drugs (benzodiazepines or benzodiazepine-like drugs) and other psychoactive drugs (antidepressants, neuroleptics, and opioid analgesics). The drug intake of both drug categories was expressed as day drugs taken/days of hospitalization for each patient.

**Table 1:** **Diagnoses on admission**.

Diagnosis	Patients (%)
Advanced/complicated cancer	12.4
Chest pain	11.3
Pulmonary embolism/COPD exacerbation	10.3
Syncope	9.3
Stroke/TIA	8.2
Heart failure	7.2
Acute pulmonary edema	5.2
Decompensated cirrhosis	5.2
Acute coronary syndrome	4.1
AF/tachyarrhythmia, bradyarrhythmia, pancreatitis	3.1
Acute anemia acute kidney failure, deep vein thrombosis, hyponatremia, intestinal occlusion, seizures, sepsis	2.1
Decompensated diabetes, hypoglycemia, jaundice	1.0

The study protocol was approved by the pertinent Institutional Committees and the study was conducted according to the Declaration of Helsinki (Hong Kong Amendment) and Good Clinical Practice (European) guidelines.

### Sleep-wake profiles

On the first day of hospitalization, pre-admission sleep-wake habits were assessed by:

- The Pittsburgh Sleep Quality Index (PSQI). This is used to assess sleep quality over the preceding month, and to differentiate “good” from “poor” sleepers. Questionnaire responses are used to generate seven components, each of which is scored from zero to three, where three represents the negative extreme. The component scores are summated to provide the PSQI global score (range: 0–21); scores of >5 identify “poor” sleepers (21, 22).
- The Epworth Sleepiness Scale (ESS). This is used to assess day-time sleepiness. Subjects rate their likelihood of “dozing off” in eight different day-time situations, on a scale of zero (unlikely), to three (very likely). The component scores are summated to provide a total score (range: 0–24); a score of ≥11 is considered abnormal (23, 24).
- The Horne–Östberg questionnaire. This is used to define diurnal preference as definitely morning (score 70–86), moderately morning (59–69), intermediate (42–58), moderately evening (31–41), and definitely evening (16–30) (25).

Daily sleep quality/timing and sleepiness during the inpatient stay were evaluated every morning between 7:30 and 8:30 by use of:

- Sleep diaries, recording bed time, sleep onset, time to fall asleep, wake-up time, get-up time, and number and duration of night awakenings. Each diary page included a Visual-Analog Scale (VAS) for assessment of sleep quality during the previous night (0 cm “bad sleep quality,” 10 cm “excellent sleep quality”) (26).
- The Karolinska Sleepiness Scale: a self-rated questionnaire, which evaluates subjective sleepiness over the previous 10 min (range 1: “very alert” – 9: “very sleepy, fighting sleep, difficulty staying awake”) (27).

### Environmental conditions

Illuminance and noise measurements were obtained four times per day at 07:30–08:30 (slot 1), 13:30–14:30 (slot 2), 18:30–19:30 (slot 3), 23:30–24:30 (slot 4, on alternate days). At the same times, information was also recorded on room lighting (central and headboard lights on/off), position of the rolling shutters (up/half-way/down), and number/type of people in the room, other than the remaining inpatients (hospital staff vs. visitors). Room lighting and rolling shutters information were coded (1/0 = light on/off; 1/0.5/0 = rolling shutters up/half-way/down), and then averaged per slot.

Illuminance was measured at eye-level for each patient, regardless of their position within the room, by a luxmeter Konica Minolta T-10 A (Konica Minolta, Marunouchi, Chiyoda, Tokyo, Japan); if the patient was not in the room, no recording was obtained.

Noise levels were obtained for five consecutive minutes (one recording per second), by use of a multifunctional PCE-222 phonometer (PCE Italia SRL, Lucca, Italy) placed on a 1.4 m height trolley in the middle of each room (Italian UNI reference 8199:1998 for rooms of approximately 20 m^2^). Noise levels were stored as average per room, and expressed in decibel A (dBA) (A-weighted decibel scale, which is adjusted for the range of normal human hearing). In addition, average noise measurements were obtained at the beginning, the middle and the end of the hallway.

### Statistical analyses

Data are presented as mean ± SD. The distribution of variables was tested for normality using the Shapiro–Wilk’s *W*-test. Differences between patients sleeping near/far from the window and in double/quadruple rooms were evaluated by the Student’s *t* or Mann–Whitney *U* test.

Differences across the 24 h and before/after the switch were analyzed by repeated measures ANOVA (*post hoc*: Scheffe test). Correlation analysis was performed by Pearson’s *r* or Spearman’s rank *R*, as appropriate.

## Results

### Sleep-wake profiles

Compliance with questionnaires and diaries was low, ranging between 2 and 59% (Table 2). This was largely related to the patients’ conditions (several were uncooperative, confused or too sick) but also to the characteristics of some of the questionnaires. Compliance was especially low (2%) for the Horne–Östberg questionnaire, which is the most complicated and the one containing questions, which are often not applicable to a population of old and diseased individuals (i.e., You have decided to do physical exercise. A friend suggests that you do this for 1 h twice a week. The best time for him/her is between 10 and 11 PM (22–23 h). Bearing in mind only your internal “clock,” how well do you think you would perform?).

**Table 2:** **Responses provided to the sleep-wake questionnaires and sleep diaries**.

	Patients (%)
Pittsburgh Sleep Quality Index	42
Epworth Sleepiness Scale	37
Horne–Östberg questionnaire	2
Karolinska Sleepiness Scale	57
Sleep diaries	59

In general, the quality of night sleep pre-hospitalization was poor, the PSQI being abnormal in 25 (60%) of the 42 patients who provided complete responses. Excessive day-time sleepiness was uncommon, the ESS being abnormal in 2 (5%) of the 37 patients who provided complete responses.

The sleep diaries kept during the inpatient stay documented reasonable subjective sleep quality (6.7 ± 2.1 on a scale of 1–10) and high sleep efficiency (hours slept/hours spent in bed: 92 ± 8%). However, 19 (33%) of the 58 subjects who completed the diaries reported that there were several nights (on average 24% of the hospitalization period) during which they had not slept at all. Reasons for sleepless nights were not reported in 44% of cases, attributed to environmental conditions in 36% (largely in relation to admission/management of other patients within the same or nearby rooms) and to worsened clinical conditions in the remaining 20% of cases (i.e., shortness of breath, chest pain, vomiting, etc.). For such nights, sleep timing parameters and the VAS scale were generally not provided, and thus not averaged. The absence of sleep timing parameters also precluded the calculation of sleep efficiency.

Average sleep onset time was 22:55 ± 1:06 and average wake-up time was 6:09 ± 0:47 (Table 3). Average, instantaneous subjective sleepiness (KSS) in the morning was low (3.5 ± 1.2 on a scale of 1 to 9). Hypnotics and psychoactive drugs were utilized for 26 ± 40% and 37 ± 45% of the days of hospitalization, respectively.

**Table 3:** **Average sleep diary variables**.

	Mean ± SD
Bed time	22:27 ± 0:58
Time to fall asleep (min)	26 ± 34
Sleep onset sleep time	22:55 ± 1:08
Wake-up time	6:09 ± 0:47
Get-up time	6:17 ± 0:43
Awakenings (*n*)	2.2 ± 2.4
Length of sleep (*h*)	7.4 ± 1.1
Sleep quality	6.7 ± 2.2
Sleep efficiency (%)	93 ± 7

Patients hospitalized before (*n* = 62) and after the switch to daylight-saving time (*n* = 37) were not significantly different in terms of age, gender, length of hospitalization, reliability in responses provided, and pre-admission sleep-wake rhythms. However, they were more commonly on sleeping medication prior to admission (PSQI component sleep medication 1.0 ± 1.4 vs. 0.3 ± 0.8, *p* < 0.05).

### Environmental conditions

Significant differences in illuminance levels between different times of the day were observed over the entire measurement period (slot 1: 182 ± 76 vs. slot 2: 481 ± 1226 vs. slot 3: 119 ± 39 vs. slot 4: 12 ± 24 lux; repeated measures ANOVA *p* < 0.001; on *post hoc* analysis, slot 2 was significantly different from each other slot). No significant differences in illuminance levels were observed between double and quadruple rooms. Significant differences in illuminance levels were observed between patients sleeping near and far from the window in slots 1 and 2 over the entire measurement period (212 ± 72 vs. 163 ± 74, *p* < 0.001; 814 ± 1876 vs. 252 ± 144, *p* < 0.05, respectively). The trend was maintained when patients were split based on being admitted before (222 ± 72 vs. 174 ± 85, *p* < 0.05; 336 ± 193 vs. 220 ± 134, *p* < 0.01, respectively) or after (198 ± 72 vs. 141 ± 37 lux, *p* < 0.01; 1460 ± 2784 vs. 318 ± 146 lux, *p* = 0.08) the switch to daylight-saving time (Table 4). No significant relationship was observed between bed position and length of hospitalization.

**Table 4:** **Light and noise measurements at different times of the day pre- and post-switch to daylight-saving time**.

	Time of day	Pre-switch	Post-switch	*p*-Value
Illuminance (lux)	07:30–08:30	192 ± 83	166 ± 62	0.13
	13:30–14:30	263 ± 167	857 ± 1972	0.02*
	18:30–19:30	114 ± 33	126 ± 46	0.16
	23:30–24:30	8 ± 20	22 ± 29	0.01*
Noise (dBA)	07:30–08:30	53 ± 3	53 ± 4	0.69
	13:30–14:30	52 ± 3	53 ± 2	0.33
	18:30–19:30	54 ± 2	54 ± 4	0.80
	23:30–24:30	45 ± 2	44 ± 3	0.17

*p < 0.05.

**Figure 1:**
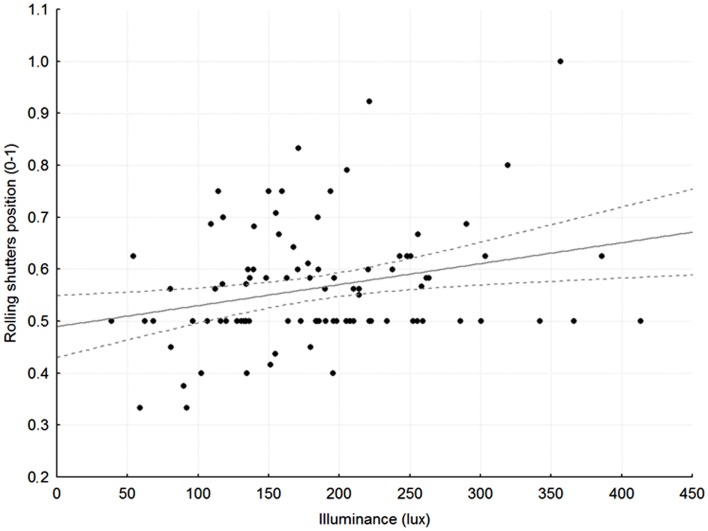
**Relationship between average rolling shutter position (1 = up, 0.5 = half-way, 0 = down) and average, recorded illuminance at patients’ eye level in slot 1, between 7:30 and 8:30 in the morning**. *r* = 0.27, *p* < 0.05; broken gray lines: 95% Confidence Intervals.

Rolling shutters were half-way down for most of the 24 h (average levels per slot 56 ± 11; 52 ± 16; 54 ± 16; 50 ± 14%, respectively). The position of the rolling shutters correlated well with luminance recorded at patients’ eye level in the early morning (slot 1: *r* = 0.26; *p* < 0.05; Figure 1) and in the late afternoon (slot 3: *r* = 0.25; *p* < 0.05).

Noise levels were comparable across time slots and constantly higher than the recommended value of 30–35 dBA for care units (Table 4). No significant relationships were observed between noise levels and type of room (double vs. quadruple), or in the rooms compared to the hallway. As for the number of people in the room other than the remaining inpatients, the highest flow was in slot 3 (range 1–4 people), the lowest in slot 4 (range 0–1 person). There was no significant relationship between the number of people in the room and recorded noise levels.

### Sleep-wake indices and environmental factors

**Figure 2:**
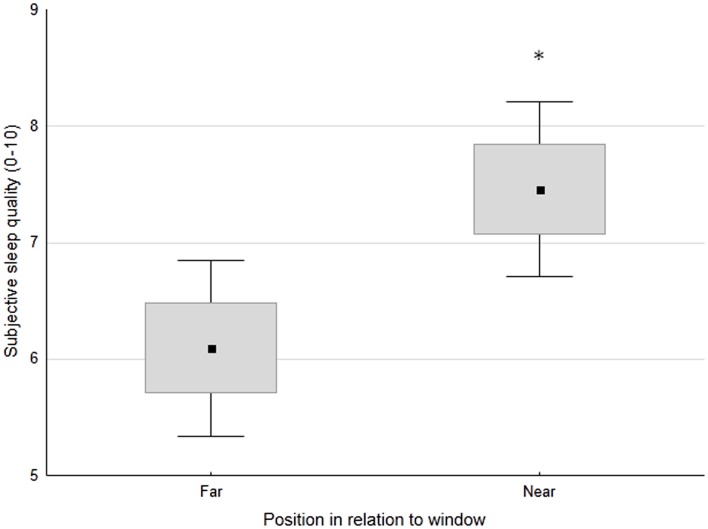
**Subjective sleep quality (0 = worst, 10 = best) in patients, classified based on the position of their bed in relation to the window (far or near)**. Small black square: mean; box: ±SE; whisker: ±1.96 SD. *p < 0.05.

No significant differences in sleep-wake profiles were observed between patients sleeping in double vs. quadruple rooms. In contrast, patients sleeping near the window had significantly better subjective sleep quality than those sleeping far from the window (7.5 ± 1.9 vs. 6.1 ± 2.2 on a scale 1–10; *p* < 0.05) (Figure 2). A similar trend was maintained both pre- and post-switch to daylight-saving time (7.3 ± 1.8 vs. 5.8 ± 2.4, *p* < 0.05; 7.7 ± 2.3 vs. 6.6 ± 1.8, n.s., respectively). As expected, given the substantially constant noise levels, no significant relationships were observed between sleep-wake profile indices and recorded noise levels.

Illuminance and noise levels pre- and post-switch are detailed in Table 4. In slot 4, illuminance was higher post-switch, most likely in relation to the central lighting system being more commonly switched on (15 ± 26 vs. 3 ± 15%, *p* < 0.05). No significant differences were observed in sleep quality or sleep timing parameters before/after the switch to daylight-saving time. The analysis of hypnotics use was confounded by differences in pre-admission habits.

## Discussion

In a large and well-characterized population of medical inpatients, compliance with sleep questionnaires and diaries was low. Patients who slept near the window were exposed to more light in the morning, and reported better subjective sleep quality. Limited variation in illuminance levels was observed across the day-time hours, and little attention was paid to light/dark hygiene, with rolling shutters being half-way down for most of the 24 h. Noise levels higher than recommended for care units and virtually constant. No changes in sleep indices were observed in relation to the switch to daylight-saving time.

### Sleep-wake assessment

Sleep-wake disturbances are common in medical inpatients. Specific pathologies such as liver failure (28), kidney failure (29, 30), stroke (31), Alzheimer’s dementia (32), and Parkinson’s disease (33, 34) have been associated with poor sleep. However, few studies have systematically analyzed sleep-wake profiles in medical inpatients by use of standard questionnaires (35–37). As a consequence, information on the applicability of standard sleep-wake assessments to medical inpatients is also lacking. The PSQI and the ESS have been validated in healthy individuals (21, 23) and in older patients with multi-pharmacological treatment (38) but not in hospitalized ones. The HÖ questionnaire has been validated only in healthy, young individuals (25). Even less formal validation work has been performed on sleep diaries/logs, of which varying formats exist and are commonly utilized in routine clinical practice (39). Difficulties are often encountered in questionnaires administration, especially in elderly individuals, who might require help in interpreting the questions, and for whom some of the questions may be inadequate with respect to their physical and/or mental conditions (40). Similar and other difficulties have been reported in specific patient populations, and it has been suggested that simplified, *ad hoc* evaluation tools may be necessary (41). In our study, compliance with sleep questionnaires and diaries was low, partly because of the characteristics of the patients enrolled, some of whom were very sick or confused, partly because of the features of the questionnaires themselves. The HÖ questionnaire proved particularly difficult, with several questions being hard to interpret and the real-life situations proposed not applicable to the patients’ routine at home (i.e., jogging for subjects with mobility issues, or working times for retired subjects). While alternative, less descriptive tools are now available and certainly worthy of formal testing, their assessment of diurnal preference still largely relies on differences between work and week-end days, thus posing problems for retired, elderly individuals (42).

Consistently with other studies and as expected based on the age of the population enrolled (21), over half of the patients who managed to complete the sleep-wake assessment reported impaired baseline sleep quality, while excessive day-time sleepiness was uncommon. During the inpatient stay, sleep diaries documented reasonable sleep quality, high sleep efficiency, and sleep times which seemed to be almost completely adjusted to hospital routine (i.e., morning cleaning, meal deliveries, and treatment administration). However, approximately a third of the subjects who provided complete diaries reported nights during which they had not slept at all; these are not captured in the diaries- and VAS-based averages, and adjustment systems may be necessary, so that sleep timing/quality can be corrected for sleepless nights, and sleep efficiency adjusted accordingly. We did not think it appropriate to apply non-validated corrections to a complex population such as the one described here but reasonable, simple solutions could be at hand (for example, a sleepless night could be described as 2*lowest sleep quality score for a single night, thus counting as two bad nights). On the other hand, it could also be argued that sleepless nights caused by worsened medical conditions and management of other patients in the same or nearby rooms (over 50% of cases in this study) should not be averaged, as they do not reflect standard inpatient sleep.

### Light

Light is the main *Zeitgeber* for synchronizing the circadian clock. Light exposure in the morning or in the evening has been proven to advance/delay circadian phase, respectively (9). Average illuminance indoor values range between 50 and 300 lux, normal office lighting provides approximately 500 lux and a bright sunny day outside providing over 100,000 lux (14, 43). As far as care facilities are concerned, a previous study documented low light exposure levels, with an average day-time illuminance of 105 lux and an average night time illuminance of 7 lux. These levels were considered insufficient for purposes of circadian entrainment (14). In addition to absolute levels, a reduced difference between day and night illuminance may weaken circadian rhythmicity, thus contributing to poor sleep. Our study showed that patients were exposed to low illuminance levels, with limited differences over the 24 h, except for the early afternoon, which was brighter, especially after daylight-saving time, and most likely in relation to improved weather conditions in April. Low illuminance levels were related, at least in part, to the fact that rolling shutters were half-way down both in the day and in the night. A significant correlation was observed between rolling shutters position and illuminance at patients’ eye level in the morning and in the late afternoon. This is reasonable, as illuminance is likely to be less dependent on shutters in the central part of the day, when it is bright outside, and at night, when it is dark. The observed relationship between shutters and illuminance in the morning and in the late afternoon also suggests that simple maneuvers such as pulling up the shutters in the early morning and pulling them down in the late afternoon might have a positive effect on entrainment, and thus on sleep quality. This hypothesis is certainly worthy of formal testing. Along the same lines, it was observed that patients sleeping near the window were exposed to more light than those sleeping far from the window, and they slept better. Given the age and the severity of the medical condition of these patients, a relatively clear effect of bed position on sleep quality was somewhat surprising. However, it is possible that in the absence of physical activity and strong food cues (disease-, treatment-, and hospitalization-related immobilization and lack of appetite), the role of light as a *Zeitgeber* and its effects on sleep quality may become more rather than less obvious. In contrast to previous reports (44), no significant relationship was observed between bed position and length of hospitalization. This is most likely due to the fact that previous reports pertained to patients with affective disorders, who tend to remain in hospital for longer than medical inpatients and in whom light impinges on the disease itself (44). In addition, there is considerable pressure to contain the duration of medical hospitalizations due to a number of reasons, to include infection transmission, hospitalization-related cognitive deterioration and economical issues. This might have masked the positive effects of bed position/light exposure, if any.

### Noise

The World Health Organization guidelines for hospital wards suggest values of ≤35 dBA in the day-time, and ≤30 dBA during the night to avoid biological, behavioral, and health effects (45). Several studies have documented far higher levels than these (4, 17, 46, 47) and both day/night noise in hospitals has increased considerably over the years [57/42 dBA in 1960 and 60/72 dBA in 2005, respectively (17)]. Dube et al. (48) surveyed a large set of intensive care patients to identify the noisiest time of the day and to identify the noise that was most disturbing to patients. The morning hours were found to be the most disturbing time, with people talking being perceived as the most disturbing source of noise. In contrast to this study, we observed noise levels, which were constantly higher than recommended but substantially comparable across time slots and little affected by the number/type of people present in the room. This, together with the absence of significant differences in noise levels between the rooms and the corridor, suggests that the main source of noise in this study was “background noise” (work and medical equipment, rather than conversations or interference from personnel/visitors). However, noise data were collected in a sparse fashion, and continuous 24-h measurements may provide a different picture. Finally, due to the fact that in rooms under 20 m^2^ noise can only be reliably measured in the middle of the room, only room and not patient-related noise levels could be obtained.

### Daylight-saving time

Finally, our study aimed at evaluating the potential influence of daylight-saving time on the sleep profile of medical inpatients. The switch to daylight-saving time in the spring had long been believed to lead to a substantially side-effect free loss of 1 h sleep on the transition night. However, more recent data suggest that increased sleep fragmentation and sleep latency present a cumulative effect of sleep loss, at least across the following week, and perhaps longer (19). Habitual sleep duration, sleep timing and diurnal preference are important predictors of the effects of the switch, with short sleepers and evening types being particularly disadvantaged in their efforts to adjust to the spring clock change (social jet lag) (49). To date, no studies have formally assessed the effect of the switch to daylight-saving time in medical inpatients. In our study, no prominent effects were detected. Hospital routine is in itself extremely disruptive: hospitalized patients spend most of their time in bed, inactive, they are forced to sleep and wake at different times compared to their routine, and are often under the effect of medications which interfere with their sleep-wake cycles. For all the above reasons, the effects of daylight-saving time on the sleep-wake cycle of medical inpatients may be masked by other, more disruptive stimuli. Alternatively, it could be argued that the absence of work/family/social day-time commitments may ease post-switch adjustment in inpatients, who are free to rest/nap in the day-time and thus may suffer less than healthy, active subjects.

In conclusion, standard sleep timing/quality questionnaires do not seem completely adequate for administration to medical inpatients, and *ad hoc* tools may be necessary. Medical wards appear to be poorly lit, constantly noisy environments, in which limited attention is paid to light/dark hygiene. The transition to daylight-saving time had limited impact on sleep-wake profiles. In contrast, a remarkable association was observed between sleep quality and bed position/light exposure, suggesting a major role for natural light on inpatient sleep quality. This association is worthy of further, formal study, and so are the effects of simple hygienic measures, such as enforced management of the rolling shutters.

## Conflict of Interest Statement

The authors declare that the research was conducted in the absence of any commercial or financial relationships that could be construed as a potential conflict of interest.
